# Long acting β_2_-adrenocepter agonists are not associated with atrial arrhythmias after pulmonary resection

**DOI:** 10.1186/s13019-017-0606-4

**Published:** 2017-05-19

**Authors:** Keiji Yamanashi, Satoshi Marumo, Ryota Sumitomo, Tsuyoshi Shoji, Motonari Fukui, Toshiro Katayama, Cheng-long Huang

**Affiliations:** 1 Department of Thoracic Surgery, Kurashiki Central Hospital, Okayama, Japan; 2 Respiratory Disease Center, Tazuke Kofukai Foundation, Medical Research Institute, Kitano Hospital, 2-4-20 Ohgimachi, Kita-ku, Osaka, 530-8480 Japan; 3 Department of Thoracic Surgery, Japanese Red Cross Otsu Hospital, Shiga, Japan; 4 Faculty of Health Sciences, Department of Medical Engineering, Himeji Dokkyo University, Hyogo, Japan

**Keywords:** Lung cancer surgery, Long-acting β_2_-adrenoceptor agonists, Postoperative respiratory complications

## Abstract

**Background:**

Long-acting β_2_-adrenoceptor agonists have been shown to increase the risk of atrial arrhythmias in patients with stable chronic obstructive pulmonary disease. The aim of this study was to investigate whether perioperative long-acting β_2_-adrenoceptor agonists treatment would increase the risk of postoperative atrial arrhythmias after lung cancer surgery in chronic obstructive pulmonary disease patients.

**Methods:**

We retrospectively analyzed 174 consecutive chronic obstructive pulmonary disease patients with non-small-cell lung cancer who underwent lobectomy or segmentectomy. The subjects were divided into those with or without perioperative long-acting β_2_-adrenoceptor agonists treatment. Postoperative cardiopulmonary complications were compared between the two groups.

**Results:**

There were no statistically significant differences between the perioperative long-acting β_2_-adrenoceptor agonists treatment group and the control group in the incidence of postoperative atrial arrhythmias (*P* = 0.629). In 134 propensity-score–matched pairs, including variables such as age, gender, comorbidities, smoking history, operation procedure, lung-cancer staging, and respiratory function, there were no significant differences between the two groups in the incidence of postoperative cardiopulmonary complications, including atrial arrhythmias.

**Conclusions:**

Perioperative administration of long-acting β_2_-adrenoceptor agonists might not increase the incidence of postoperative atrial arrhythmias after surgical resection for non-small-cell lung cancer in chronic obstructive pulmonary disease patients.

**Electronic supplementary material:**

The online version of this article (doi:10.1186/s13019-017-0606-4) contains supplementary material, which is available to authorized users.

## Background

Chronic obstructive pulmonary disease (COPD) is primarily characterized by the presence of airflow limitation resulting from parenchymal destruction (emphysema) and airway remodeling [1]. Therefore, the mainstay of pharmacological treatment in stable COPD are bronchodilators, such as long-acting β_2_-adrenoceptor agonists (β_2_-agonists) and long-acting muscarinic antagonists [2]. These bronchodilators have been shown to improve symptoms, quality-of-life, pulmonary function and mortality in patients with COPD.

COPD cases are often complicated by lung cancer, because both conditions are strongly associated with cigarette smoking [3]. With the improvement in mortality from COPD itself due to bronchodilators, lung cancer has come to be one of the most important problems in COPD. Surgical operation is the first-choice treatment in COPD patients with resectable lung cancer. However, atrial arrhythmias often occur as a postoperative complication following thoracic surgery [4] and may be associated with an increased risk of cerebral embolism, since the administration of anticoagulation therapy must be balanced against the risk of bleeding. Therefore, postoperative atrial arrhythmias should be avoided in COPD patients with lung cancer who undergo surgical resection.

There are several reasons why atrial arrhythmias often occur after surgical operation for lung cancer in COPD patients. One is that COPD itself is a risk factor for postoperative atrial arrhythmias [5]. Another reason is that lung cancer surgery also entails a risk of postoperative atrial arrhythmias. According to recent studies, the arrhythmias result from the synergic action of increased vagal tone, atrial inflammation, pulmonary hypertension, right heart strain, hypoxemia, and anatomical substrate, such as surgical damage to the cardiac plexus or to the proximal trunks of the pulmonary veins [6, 7]. Moreover, several reports have shown that β_2_-agonists increase the risk of atrial arrhythmias in COPD patients who do not undergo surgery [8, 9]. However, the effects of β_2_-agonists treatment during the perioperative period on postoperative atrial arrhythmias after lung cancer surgery have not been elucidated.

The aim of this study was to investigate whether perioperative β_2_-agonists treatment would increase the risk of postoperative atrial arrhythmias after pulmonary resection for non-small-cell lung cancer (NSCLC) in chronic obstructive pulmonary disease patients.

## Methods

### Patient selection

We conducted a retrospective analysis of the COPD patients diagnosed with NSCLC who underwent surgery at the Tazuke Kofukai Medical Research Institute, Kitano Hospital, between January 2007 and December 2014. The exclusion criteria were as follows: no pathological confirmation of NSCLC, lesser resection (wedge resection), repeated pulmonary resection, pneumonectomy, chronic atrial arrhythmias before surgery, evidence of infection such as pneumonia before surgery, thyroid dysfunction, and renal failure requiring hemodialysis [10–12]. COPD was diagnosed on the basis of the Global Initiative for Chronic Obstructive Lung Disease (GOLD) [13]. The results of the perioperative β_2_-agonists treatment group (β_2_-agonists group) and non-β_2_-agonists treatment group (control group) were compared and analyzed regarding postoperative cardiopulmonary complications. The patients were on continuous electrocardiogram monitoring from surgery day to a week after surgery. After a week, we evaluate postoperative cardiopulmonary complications by intermittent monitoring and examinations. In the perioperative β_2_-agonists treatment group, the subjects received tulobuterol tape (2 mg/day), inhaled indacaterol (150 μg/day), or inhaled salmeterol (100 μg/day) from more than 2 weeks before surgery until at least a month after surgery, without interruption. The data from the respiratory function tests performed before and after perioperative β_2_-agonists treatment were compared in the few cases where such data were tracked.

Study approval was granted by the ethics committee of the Tazuke Kofukai Medical Research Institute, Kitano Hospital, in accordance with the Declaration of Helsinki.

### Surgical procedure

All patients underwent lobectomies or segmentectomies with anterolateral thoracotomy, posterolateral thoracotomy, or video-assisted thoracic surgery (VATS). For VATS, three access ports were placed through 2–3 cm axillary skin incisions. One of these incisions was extended by 4–5 cm, and the resected lung was removed in a plastic bag without using a rib spreader. Patients requiring conversion from VATS to thoracotomy were classified as open thoracotomy patients.

### Postoperative cardiopulmonary complications

All patients were followed-up after surgery, and complications occurring during the same hospitalization as the index procedure were recorded. Cardiopulmonary complications were defined as previously described [10] and included cardiovascular complications, such as arrhythmias (atrial fibrillation [AF], paroxysmal supraventricular tachycardia [PSVT], ventricular tachycardia [VT]), angina pectoris, acute myocardial infarction (AMI), congestive heart failure (CHF), thromboembolic events; and respiratory complications such as pneumonia (fever >38 °C, purulent sputum, abnormal findings on chest X-ray), atelectasis with bronchoscopic therapy, acute respiratory distress syndrome (ARDS) (partial pressure of oxygen in arterial blood-fraction of inspired oxygen <300 mmHg), respiratory insufficiency requiring tracheostomy, and respiratory failure requiring mechanical ventilation. As prolonged air leak and bronchopleural fistulas are considered surgical factors, they were excluded.

### Endpoints

The primary endpoint was the incidence of postoperative atrial arrhythmias (AF and PSVT) after surgical resection. Secondary endpoint was the incidence of the other cardiopulmonary complications after surgical resection.

### Statistical analysis

The data are presented as mean ± standard deviation. Categorical variables are shown as percentages of the sample. Continuous variables were compared using the Welch’s *t* test and categorical variables using the Fisher’s exact test or chi-squared test. Propensity score matching was applied to balance the assignment of patients for correct evaluation of the effects of β_2_-agonists treatment during the perioperative period. The variables were age, gender, comorbidities, smoking history, operation procedure, lung-cancer staging, and respiratory function. Univariate logistic regression analyses were performed for postoperative atrial arrhythmias to observe Type 1 error. We assessed the time free of postoperative atrial arrhythmias using Kaplan–Meier analysis. Differences between term curves were tested for statistical significance using the two-tailed log-rank test. All data were processed and analyzed using SPSS version 20.0 (SPSS, Chicago, IL, USA) or the statistical software R version 3.0.3 (R Foundation for statistical computing, Vienna, Austria). All *P*-values are 2-sided, and *P*-values < 0.1 were considered statistically significant.

## Results

### Subjects

**Fig. 1:**
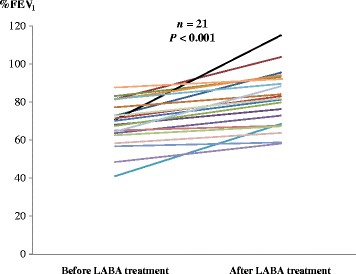
A comparison of percentage forced expiratory volume in 1 s (%FEV_1_) before and after perioperative treatment with long-acting β_2_-adrenoceptor agonists (β_2_-agonists). %FEV_1_ improved significantly after perioperative β_2_-agonists treatment (*P* < 0.001)

Data from the 191 COPD patients diagnosed with NSCLC who underwent surgery at our hospital between January 2007 and December 2014 were obtained from the hospital’s database. Seventeen patients were excluded because of lesser resection (wedge resection) (*n* = 4), pneumonectomy (*n* = 8), and chronic atrial arrhythmias before surgery (*n* = 5). Thus, 174 patients were included in this study. The clinicopathological characteristics of patients are shown in Table 1. There were no significant differences in age, gender, comorbidities, smoking history, surgical procedure, or lung cancer staging between the perioperative β_2_-agonists group and the control group. However, there were statistically significant differences between the two groups in forced expiratory volume in 1 s (FEV_1_) and the ratio of FEV_1_ to forced vital capacity (FVC). Furthermore, %FEV_1_ after administration of β_2_-agonists was significantly increased compared with %FEV_1_ before administration of β_2_-agonists (Fig. 1).

**Table 1:** Characteristics of patients with chronic obstructive pulmonary disease

	β_2_-agonists group (*n* = 71)	Control group (*n* = 103)	*P*-value
Age, years	71.4 ± 7.3	70.7 ± 8.6	0.523
Gender (male)	55 (76%)	80 (78%)	0.975
Comorbidities (HT/DL/DM/IHD)	20/8/9/3	29/15/15/7	NS
Smoking history	65 (92%)	93 (90%)	0.778
Surgical procedure (VATS)	26 (37%)	49 (48%)	0.152
Lung cancer staging (I/II/III/IV)	48/8/13/2	68/9/22/4	NS
VC, % predicted	94.9 ± 14.6	98.0 ± 16.7	0.186
FEV_1_, % predicted	69.6 ± 15.1	79.0 ± 17.0	<0.001
FEV_1_/FVC, %	57.9 ± 8.6	62.9 ± 6.8	<0.001

Values are shown as numbers (%) or mean ± SD

Abbreviations: *DL* dyslipidemia, *DM* diabetes mellitus, *FEV*_*1*_ forced expiratory volume in 1 s, *FVC* forced vital capacity, *HT* hypertension, *IHD* ischemic heart disease, *NS* not significant, *VATS* video-assisted thoracoscopic surgery, *VC* vital capacity

### Postoperative cardiopulmonary complications

Postoperative cardiopulmonary complications are shown in Table 2. Atrial arrhythmias (AF and PSVT) were the most frequent complication. There were no events of VT, angina pectoris, CHF, or ARDS. There were no statistically significant differences between the two groups in the incidence of cardiovascular complications, AF, PSVT, AMI, thromboembolic events, respiratory complications, pneumonia, atelectasis, respiratory insufficiency, or respiratory failure.

**Table 2:** Postoperative cardiopulmonary complications

	β_2_-agonists group (*n* = 71)	Control group (*n* = 103)	*P*-value
Cardiovascular complications	7 (10%)	10 (10%)	0.974
Atrial arrhythmias	7 (10%)	8 (8%)	0.629
Atrial fibrillation	7 (10%)	7 (7%)	0.465
Paroxysmal supraventricular tachycardia	0	1 (1%)	0.405
Acute myocardial infarction	0	1 (1%)	0.405
Thromboembolic events	0	2 (2%)	0.238
Respiratory complications	9 (13%)	11 (11%)	0.685
Pneumonia	5 (7%)	5 (5%)	0.542
Atelectasis with bronchoscopic therapy	4 (6%)	4 (4%)	0.588
Respiratory insufficiency requiring tracheostomy	0	1 (1%)	0.405
Respiratory failure requiring mechanical ventilation	1 (1%)	3 (3%)	0.515

### Evaluation of postoperative atrial arrhythmias between perioperative β_2_-agonists treatment group and control group using propensity score-matched analysis

**Fig. 2:**
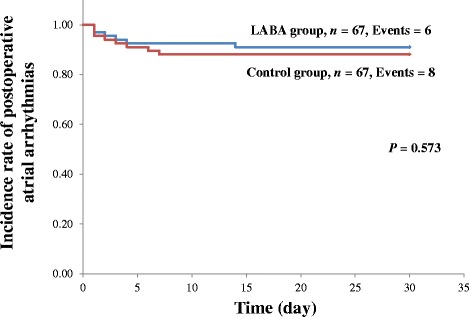
Kaplan–Meier analyses of the incidence of postoperative atrial arrhythmias in the patients after propensity score matching, stratified by perioperative treatment with long-acting β_2_-adrenoceptor agonists (β_2_-agonists). There were no statistically significant differences between the two groups in the time free of postoperative atrial arrhythmias (*P* = 0.573)

Propensity score matching was used and variables such as age, gender, comorbidities, smoking history, operation procedure, lung cancer staging, and respiratory function were included, because it was possible that these factors affected the incidence of postoperative cardiopulmonary complications [11]. The perioperative β_2_-agonists treatment group and control group were well matched (67 patients each), without significant differences in clinical factors (Table 3). The effect size and bias reduction were shown in Table 4. The effect sizes and the mean bias reduction were well validated. There were no statistically significant differences between the two groups in the limited analysis of patients with cardiopulmonary complications including atrial arrhythmias (Table 5). Univariate logistic regression analyses showed there were no statistically significant associations between atrial arrhythmias and other cardiopulmonary complications (Additional file 1: Table S1). Kaplan–Meier analysis was performed to determine whether perioperative β_2_-agonists treatment had an effect on postoperative atrial arrhythmias after lung cancer surgery in the COPD patients. There were no statistically significant differences between the two groups in the time free of postoperative atrial arrhythmias (*P* = 0.573; Fig. 2).

**Table 3:** Propensity score-matched comparison of clinical factors

Variables	β_2_-agonists group (*n* = 67)	Control group (*n* = 67)	*P*-value
Age, years	71.4 ± 7.4	70.5 ± 8.5	0.503
Gender (male)	53 (79%)	52 (78%)	0.834
Comorbidities (HT/DL/DM/IHD)	19/8/9/3	18/9/10/3	NS
Smoking history	61 (91%)	59 (88%)	0.572
Surgical procedure (VATS)	22 (33%)	29 (43%)	0.213
Lung cancer staging (I/II/III/IV)	46/7/12/2	42/6/16/3	NS
VC, % predicted	95.7 ± 14.2	97.6 ± 16.9	0.475
FEV_1_, % predicted	70.6 ± 14.9	75.0 ± 17.0	0.108
FEV_1_/FVC, %	58.2 ± 8.6	60.2 ± 7.0	0.145

Values are shown as numbers (%) or mean ± SD

Abbreviations as in Table 1

**Table 4:** The effect size and bias reduction

	β_2_-agonists group	Control group					
Logistic regression analysis							
	*n* = 71	*n* = 103	Difference of means	Standard error	*P*-value	Effect size	Bias reduction
Age	71.4 ± 7.3	70.7 ± 8.6	0.7	0.86	0.52	0.82	
VC, % predicted	94.9 ± 14.6	98.0 ± 16.7	−3.1	1.69	0.19	1.84	
FEV_1_, % predicted	69.6 ± 15.1	79.0 ± 17.0	−9.4	1.73	<0.001	5.42	
FEV_1_/FVC, %	57.9 ± 8.6	62.9 ± 6.8	−5.0	0.85	<0.001	5.92	
Logistic regression analysis adjusted propensity score							
	*n* = 67	*n* = 67					
Age	71.4 ± 7.4	70.5 ± 8.5	0.9	0.97	0.50	0.93	113.4
VC, % predicted	95.7 ± 14.2	97.6 ± 16.9	−1.9	1.90	0.47	1.00	54.5
FEV_1_, % predicted	70.6 ± 14.9	75.0 ± 17.0	−4.4	1.95	0.11	2.26	41.6
FEV_1_/FVC, %	58.2 ± 8.6	60.2 ± 7.0	−2.0	0.95	0.14	2.10	35.5
Mean of bias reduction							61.3

*Abbreviations*: *FEV*_*1*_ forced expiratory volume in 1 s, *FVC* forced vital capacity, *VC* vital capacity

**Table 5:** Postoperative cardiopulmonary complications using propensity score-matched analysis

Variables	β_2_-agonists group (*n* = 67)	Control group (*n* = 67)	*P*-value
Cardiovascular complications	6 (9%)	9 (13%)	0.411
Atrial arrhythmias	6 (9%)	8 (12%)	0.572
Atrial fibrillation	6 (9%)	7 (10%)	0.770
Paroxysmal supraventricular tachycardia	0	1 (2%)	0.316
Acute myocardial infarction	0	1 (2%)	0.316
Thromboembolic events	0	1 (2%)	0.316
Respiratory complications	9 (13%)	8 (12%)	0.795
Pneumonia	5 (8%)	3 (5%)	0.466
Atelectasis with bronchoscopic therapy	4 (6%)	4 (6%)	1.000
Respiratory insufficiency requiring tracheostomy	0	0	1.000
Respiratory failure requiring mechanical ventilation	1 (2%)	1 (2%)	1.000

### Power calculation

We calculated that a two-tailed *t* test with a 10% significance level and 80% power would require 2095 patients for the primary endpoint of the present study. Therefore, 174 patients (β_2_-agonists group: 71 and control group: 103, and incidence of atrial arrhythmias of the subjects β_2_-agonists group: 7 and control group: 8) in the present study were considered low power.

## Discussion

In this retrospective observational study, the perioperative administration of β_2_-agonists did not increase the incidence of postoperative atrial arrhythmias (AF and PSVT) after surgical resection for NSCLC in patients with COPD. The incidence of other postoperative cardiopulmonary complications such as VT, angina pectoris, AMI, CHF, thromboembolic events, pneumonia, atelectasis, ARDS, respiratory insufficiency and respiratory failure was also not increased by the perioperative administration of β_2_-agonists.

β_2_-agonists are among the first-choice drugs for the treatment of patients with stable COPD [2]. Several reports have shown that treatment with β_2_-agonists increased the risk of atrial arrhythmias in stable COPD patients [8, 9]. The mechanism involves β_2_-agonists’s stimulation of the β_1_-adrenoceptor of the cardiac conduction system. β_2_-agonists are usually administered using inhaler devices and have high selectivity for the β_2_-adrenoceptor [14]. However, they can cause adverse systemic effects, such as atrial arrhythmias, by migrating from lung to blood and stimulating the β_1_-adrenoceptor of the cardiac conduction system.

On the other hand, β_2_-agonists may have various beneficial effects on atrial arrhythmias. First, postoperative atrial arrhythmias are considered to result from right heart strain [6, 7]. β_2_-agonists have a strong bronchodilation effect that leads to amelioration of right heart strain by modifying the hyperinflation in patients with stable COPD [15]. Therefore, the bronchodilation of β_2_-agonists may reduce the risk of atrial arrhythmias in patients with stable COPD. Second, in the perioperative period, a low predicted postoperative FEV_1_ has been shown to be the best indicator of patients at high risk for pulmonary resection surgery [16]. Suzuki et al. showed that significant FEV_1_ improvement was observed after the use of perioperative bronchodilator treatment in lung cancer patients with COPD [17]. In the present study, %FEV_1_ improved significantly after perioperative β_2_-agonists treatment in the few cases whose data was tracked (*P* < 0.001; Fig. 1). Therefore, perioperative β_2_-agonists treatment might improve FEV_1_, resulting in a protective effect against postoperative cardiopulmonary complications such as atrial arrhythmias. These harmful and beneficial effects of β_2_-agonists on atrial arrhythmias might cancel each other out. In the present study, the perioperative administration of β_2_-agonists did not increase the incidence of postoperative atrial arrhythmias after surgical resection for NSCLC in patients with COPD.

The incidence rate of atrial arrhythmias after pulmonary resection in the present study was about 9%. Compared with other studies (10–12%) [18, 19], this rate was a little low. Because the patients having risk factors of atrial arrhythmias were excluded in the criteria, low incidence rate of atrial fibrillation might be found.

We acknowledge limitations of the present study. First, the present study was retrospective. Second, the present study was limited by being merely a small sample size and low power to investigate the effect of β_2_-agonists on atrial arrhythmias, rather than a randomized controlled interventional trial. To compensate for these limitations, we performed a propensity score matching analysis. These analyses indicated that perioperative β_2_-agonists treatment was not correlated with the risk of postoperative atrial arrhythmias in COPD patients with NSCLC.

## Conclusions

This study showed that the association between perioperative β_2_-agonists treatment and the risk of postoperative atrial arrhythmias after pulmonary resection for NSCLC in COPD patients. Perioperative administration of β_2_-agonists might not increase the incidence of postoperative atrial arrhythmias after surgical resection for NSCLC in COPD patients. Further prospective studies with a larger number of patients from multiple institutions will be required to confirm the present findings.

## Additional file

Additional file 1: Table S1. Univariate logistic regression analysis for postoperative atrial arrhythmias after propensity score-matched analysis (DOCX 17 kb)

## Abbreviations

AF: Atrial fibrillation
AMI: Acute myocardial infarction
ARDS: Acute respiratory distress syndrome
CHF: Congestive heart failure
COPD: Chronic obstructive pulmonary disease
FEV_1_: Forced expiratory volume in 1 s
FVC: Forced vital capacity
GOLD: Global Initiative for Chronic Obstructive Lung Disease
NSCLC: Non-small-cell lung cancer
PSVT: Paroxysmal supraventricular tachycardia
VATS: Video-assisted thoracoscopic surgery
VT: ventricular tachycardia
