# Supplementary material for: Long acting β2-adrenocepter agonists are not associated with atrial arrhythmias after pulmonary resection
Source: J Cardiothorac Surg. 2017 May 19;12:35. doi: 10.1186/s13019-017-0606-4 (PMC5437531; doi:10.1186/s13019-017-0606-4)
Supplement: Additional file 1: Table S1. — Univariate logistic regression analysis for postoperative atrial arrhythmias after propensity score-matched analysis (DOCX 17 kb) [file 13019_2017_606_MOESM1_ESM.docx]

Table S1. Univariate logistic regression analysis for postoperative atrial arrhythmias after propensity score-matched analysis

|  |  | Incidence Rate | Univariate logistic regression analysis | | |
| --- | --- | --- | --- | --- | --- |
|  |  |  | OR | 95% CI | *P*-value |
| Acute myocardial infarction | - | 133 (99%) | reference |  |  |
|  | + | 1 (1%) | < 0.001 | - | 0.993 |
| Thromboembolic events | - | 133 (99%) | reference |  |  |
|  | + | 1 (1%) | > 1000 | - | 0.990 |
| Pneumonia | - | 126 (37%) | reference |  |  |
|  | + | 8 (63%) | 1.240 | 0.140 – 10.900 | 0.845 |
| Atelectasis with bronchoscopic therapy | - | 126 (37%) | reference |  |  |
|  | + | 8 (63%) | 1.240 | 0.141 – 10.900 | 0.845 |
| Respiratory insufficiency requiring tracheostomy | - | 134 (100%) | reference |  |  |
|  | + | 0 (0%) | - | - | - |
| Respiratory failure requiring mechanical ventilation | - | 132 (99%) | reference |  |  |
|  | + | 2 (1%) | < 0.001 | - | 0.993 |

Abbreviations: CI, confidence interval; OR, odds ratio.
